# *Helicobacter pylori* infection increases risk of incident metabolic syndrome and diabetes: A cohort study

**DOI:** 10.1371/journal.pone.0208913

**Published:** 2019-02-19

**Authors:** Yuan-Yuei Chen, Wen-Hui Fang, Chung-Ching Wang, Tung-Wei Kao, Yaw-Wen Chang, Chen-Jung Wu, Yi-Chao Zhou, Yu-Shan Sun, Wei-Liang Chen

**Affiliations:** 1 Department of Internal Medicine, Tri-Service General Hospital Songshan Branch, and School of Medicine, National Defense Medical Center, Taipei, Taiwan, Republic of China; 2 Division of Family Medicine, Department of Family and Community Medicine, Tri-Service General Hospital, and School of Medicine, National Defense Medical Center, Taipei, Taiwan, Republic of China; 3 Division of Geriatric Medicine, Department of Family and Community Medicine, Tri-Service General Hospital, and School of Medicine, National Defense Medical Center, Taipei, Taiwan, Republic of China; 4 Health Management Center, Department of Family and Community Medicine, Tri-Service General Hospital, National Defense Medical Center, Taipei, Taiwan, Republic of China; 5 Division of Family Medicine, Department of Community Medicine, Taoyuan Armed Forces General Hospital, Taoyuan, Taiwan, Republic of China; China Medical University, TAIWAN

## Abstract

Emerging studies have shed light on the association between *Helicobacter pylori* (HP) infection and cardiometabolic risk. However, there is no evidence to support a causal link for the relationship in the general population. Our aim was to determine whether HP infection is associated with the risks of incident type II diabetes mellitus (DM) in a population-based cohort consisting of adults from the general population. A total of 69235 adults enrolled in the study obtained health examinations at the Tri-Service General Hospital in Taiwan from 2010 to 2016. HP infection detection was performed by rapid urease tests (RUTs), and endoscopic examinations were used to diagnose gastroesophageal reflux disease (GERD), gastric ulcers (GUs) and duodenal ulcers (DUs). Cross-sectional and longitudinal analyses were performed to examine the association between HP infection and cardiometabolic diseases using logistic regression and Cox regression in a large population-based study. HP infection was significantly associated with the presence of metabolic syndrome (MetS) (OR = 1.26, 95%CI: 1.00–1.57) and DM (OR = 1.59, 95%CI: 1.17–2.17) only in male subjects, and abnormal endoscopic findings were also correlated with cardiometabolic diseases. Our findings demonstrated that participants with HP infection had an elevated risk of developing incident DM (HR = 1.54, 95%CI: 1.11–2.13). In addition, endoscopic findings of a DU (HR = 1.63, 95%CI: 1.02–2.63), rather than GERD or a GU, were also predictive of incident DM. In this cohort, HP infection was a statistically significant predictor of incident DM among male population.

## Introduction

*Helicobacter pylori* (HP) is a common worldwide infection in developing countries and results in gastrointestinal complications such as peptic ulcer and gastric cancer[1]. HP can affect a host organism mainly by the proteins cytotoxin-associated gene A (CagA) and vacuolating cytotoxin A (VacA)[2], which disturb host immunological responses and cause the release of pro-inflammatory cytokines[3]. Thus, prevalent extra-gastroduodenal diseases have been reported in those with HP infection including coronary heart disease[4], hepatobiliary diseases[5], and autoimmune disorders[6].

Type II diabetes mellitus (DM) has become an emerging health problem in recent decades. The pathophysiology of DM is complicated, with risk factors correlated to lifestyle and genetic background[7]. Simon et al. first suggested the link between HP infection and DM[8], and the role of HP in the development of DM has since become better understood. Considerable evidence indicates that the pathogenic mechanisms for DM such as inflammation and insulin resistance are induced by HP infection[9].

In a previous study examining 782 elderly adults, HP infection led to an increased rate of incident DM in a cohort study[10]. However, there is no evidence to support a causal link for the relationship in the general population. Our aim was to determine whether HP infection is associated with the risks of incident DM in a population-based cohort consisting of adults from the general population.

## Methods

### Study design

**Fig 1:**
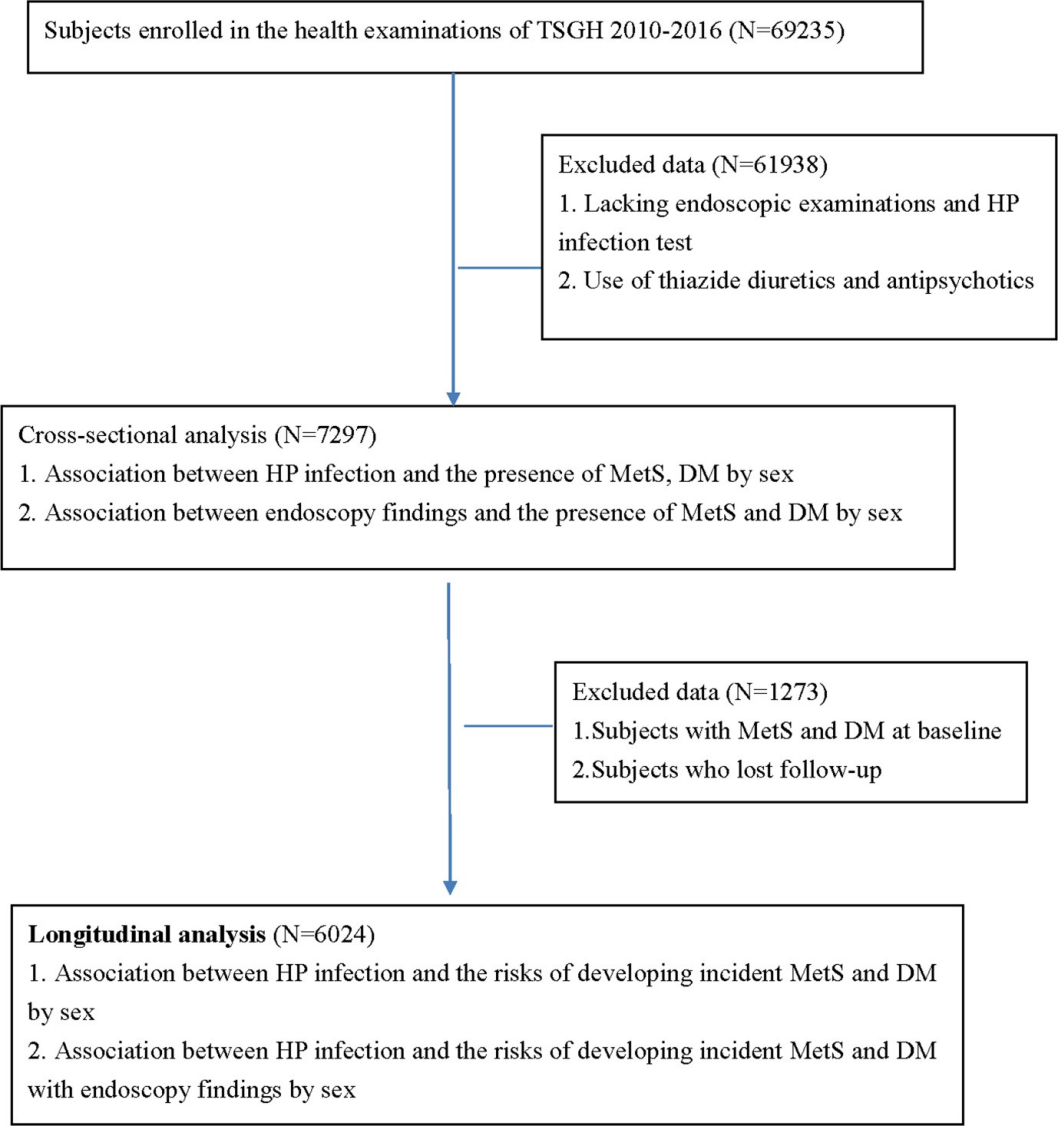
Flow chart of our study.

Data were obtained from individuals aged 20 years old and older enrolled in a retrospective study from health examinations at Tri-Service General Hospital from 2010 to 2016. Study approval was conduct by the Institutional Review Board of Tri-Service General Hospital, Taiwan. According to the flow chart of our study shown in Fig 1, 69235 subjects were enrolled in the health check-up during this period. Exclusion criteria included use of thiazide diuretics and antipsychotics, and those lacking endoscopy examinations and HP infection tests. A total of 7297 eligible subjects were analyzed in a cross-sectional study. First, tests for sex differences in odds ratios (ORs) for associations between HP infection, MetS and DM were performed. Second, tests for sex differences in ORs for associations between gastroesophageal reflux disease (GERD), gastric ulcer (GU), duodenal ulcer (DU), MetS and DM were conducted. In the next step, subjects who visited the health examination twice were included and we excluded those with MetS and DM at baseline and lost to follow-up. A longitudinal analysis composed of 6024 participants over an average of 7.2 years of follow-up was performed. Hazard ratios (HRs) of HP infection for predicting incident MetS and DM were calculated by the Cox proportional hazard model. In addition, HRs of HP infection for predicting MetS and DM with endoscopy findings by sex were also examined.

### Diagnosis of HP infection

Traditional endoscopic exams were performed during the health check-up to diagnose HP infection[11]. As a standard and accurate examination, it was also commonly applied in the diagnosis of HP-associated diseases including peptic ulcer diseases, mucosa-associated lymphoid tissue lymphoma and gastric cancer. A rapid urease test (RUT), the most useful noninvasive test for detecting HP infection, was performed in the study because it is affordable, simple, highly specific and frequently used in clinical practice[12]. The basis of the test is the ability of HP to catalyze the conversion of urea to ammonia and carbon dioxide by the urease enzyme, which raises the pH and changes the color of the pH monitor. The Pronto Dry, a new rapid urease test, with a higher sensitivity in the pre- and post- treatment setting than that of the liquid phase-rapid urease test, was used in the health examinations in our study[13].

### Definition of MetS

The diagnosis criteria of MetS were met if an individual ≥ 3 components as follows based in the Taiwan Health Promotion Administration of the Ministry of Health and Welfare in 2007: (1) waist circumference: male>90 cm and female>80 cm; (2) systolic blood pressure (SBP) ≥ 130 mmHg or diastolic blood pressure (DBP) ≥ 80 mmHg, or self-reported hypertension; (3) triglycerides ≥ 150 mg/dL (1.7 mmol/L); (4) fasting plasma glucose ≥ 100 mg/dL, or a past history of a diabetic status; (5) high density lipoprotein cholesterol (HDL-C): male<40 mg/dL (1.03 mmol/L) and female<50 mg/dL (1.3 mmol/L).

### Definition of DM

Following the criteria of the American Diabetes Association, subjects with one of the following components were diagnosed with DM: (1) fasting plasma glucose≥126 mg/dL, (2) hemoglobin A1c test: ≥6.5%, (3) oral glucose tolerance test (OGTT): ≥200 mg/dL, (4) random plasma glucose: ≥200 mg/dL, (5) past history of a diabetic status, or use of antidiabetic agents[14]. The first 4 items should be rechecked at least twice.

### Covariates measurement

Body mass index (BMI) was estimated based on a general formula in which the weight in kilograms was divided by the square of the height in meters (kg/m^2^) of a participant. Biochemical laboratory data were collected by drawing blood samples from subjects after fasting for at least 8 hours and analyzed by different standard procedures. Cigarette smoking was obtained from participants by asking a question “How many packs do you smoke per day?”. Alcoholic drinking was determined by self-report questionnaire dividing into ‘‘never” and ‘‘alcohol consumption”.

### Statistical analysis

Statistical Package for the Social Sciences, version18.0 (SPSS Inc., Chicago, IL, USA) for Windows was used for statistical analyses in the study. The differences between males and females in terms of demographic information and biochemical data were examined by Student’s t test and Pearson's chi-square test. The threshold for statistical significance was defined by a two-sided *p*-value of ≤ 0.05. An extended-model approach was performed in the study with multivariable adjustment for pertinent clinical variables. For cross-sectional analysis, multivariate logistic regression was conducted for the associations among HP infection, endoscopic findings and the cardiometabolic risks in the male and female groups (Model 1: unadjusted; Model 2: Model 1 + age, BMI, proteinuria, low density lipoprotein cholesterol (LDL-C), uric acid (UA), creatinine (Cr), aspartate aminotransferase (AST), albumin, high sensitivity C-reactive protein (hsCRP), thyroid stimulating hormone (TSH); Model 3: Model 2 + history of smoking, drinking). For longitudinal analysis, multivariate Cox regression was performed for the associations of HP infection and incident MetS, DM, prediabetes and HTN (Model 1: unadjusted; Model 2: Model 1 + age, BMI, proteinuria, serum total cholesterol, uric acid, creatinine, AST, albumin, hsCRP, TSH; Model 3: Model 2 + history of smoking, drinking).

## Results

### The demographic characteristics of the study sample

The characteristics of the study population are listed in Table 1. The mean ages of the males and females were 51.61±12.29 and 50.73±11.86 years old, respectively. The prevalence of HP infection was 18.8% in males and 15.1% in females in the study. Most characteristics including BMI, components of MetS, laboratory biochemical data and past histories except proteinuria, had significant differences.

**Table 1:** Characteristics of study sample.

Variables	Male (N = 3757)	Female (N = 2874)	P Value
**Continuous Variables, mean (SD)**			
**Age (years)**	51.61 (12.29)	50.73 (11.86)	0.003
**BMI (kg/m**^**2**^**)**	25.19 (3.44)	22.93 (3.70)	<0.001
**LDL-C (mg/dL)**	122.58 (33.37)	118.62 (32.65)	<0.001
**UA (mg/dL)**	6.44 (1.33)	4.82 (1.15)	<0.001
**Cr (mg/dL)**	0.97 (0.32)	0.68 (0.20)	<0.001
**AST (U/L)**	23.99 (12.61)	21.05 (10.03)	<0.001
**Albumin (g/dL)**	4.51 (0.26)	4.42 (0.26)	<0.001
**hsCRP (mg/dL)**	0.26 (0.58)	0.22 (0.40)	<0.001
**TSH (uIU/mL)**	2.19 (1.69)	2.53 (1.89)	<0.001
**Category Variables, (%)**			
**HP infection**	708 (18.8)	433 (15.1)	<0.001
**Smoking**	1852 (51.1)	295 (10.8)	<0.001
**Drinking**	2093 (65.9)	715 (29.2)	<0.001

BMI, body mass index; LDL-C, low density lipoprotein cholesterol; UA, uric acid; Cr, creatinine; AST, aspartate aminotransferase; hsCRP, high sensitivity C-reactive protein; TSH, thyroid stimulating hormone

### Relationship between HP infection and MetS and DM

The associations between HP infection and cardiometabolic diseases by sex were analyzed by logistic regression, and the results are shown in Table 2. After fully adjusting for covariables, HP infection had a positive association with MetS and DM in males with ORs of 1.25 (95%CI = 1.01–1.55) and 1.53 (95%CI = 1.15–2.04), respectively. However, no significant differences were noted in female participants.

**Table 2:** Association between HP infection and the presence of MetS, DM categorized by gender.

	Sex	Model ^a^ 1 OR (95% CI)	*P* Value	Model ^a^ 2 OR (95% CI)	*P* Value	Model ^a^ 3 OR (95% CI)	*P* Value
	**MetS**						
**HP infection**	**Male**	1.30 (1.08–1.57)	0.006	1.27 (1.03–1.57)	0.028	1.25 (1.01–1.55)	0.042
	**Female**	1.05 (0.80–1.37)	0.754	0.73 (0.53–1.00)	0.052	0.73 (0.53–1.01)	0.055
	**DM**						
**HP infection**	**Male**	1.74 (1.31–2.30)	<0.001	1.56 (1.17–2.08)	0.003	1.53 (1.15–2.04)	0.004
	**Female**	1.05 (0.62–1.79)	0.853	0.84 (0.48–1.46)	0.526	0.83 (0.47–1.45)	0.512

^a^ Adjusted covariates

Model 1 = unadjusted

Model 2 = Model 1 + age, BMI, LDL-C, hsCRP

Model 3 = Model 2 + history of smoking, drinking

### Association between endoscopic findings and the presence of MetS and DM

In our study, participants with different diagnoses of GERD, GU and DU as determined by endoscopy were analyzed to predict the presence of MetS and DM. The results are summarized in Table 3. In males, GERD had a significant relationship with the presence of MetS and DM with ORs of 1.28 (95%CI = 1.02–1.59) and 1.68 (95%CI = 1.14–2.48) in the fully adjusted model. GU had a significant relationship with the presence of MetS with ORs of 1.77 (95%CI = 1.20–2.61). DU had a significant relationship with the presence of DM with ORs of 2.10 (95%CI = 1.22–3.59). However, female participants who underwent endoscopic examinations had no significant association between endoscopic findings and adverse health outcomes.

**Table 3:** Association between endoscopy findings and the presence of MetS and DM categorized by gender.

	Variable	Model ^a^ 1 OR (95% CI)	*P* Value	Model ^a^ 2 OR (95% CI)	*P* Value	Model ^a^ 3 OR (95% CI)	*P* Value
**Male**	**MetS**						
	**Normal**	Reference	-	Reference	-	Reference	-
	**GERD**	1.47 (1.21–1.78)	<0.001	1.33 (1.07–1.66)	0.011	1.28 (1.02–1.59)	0.031
	**GU**	2.04 (1.45–2.86)	<0.001	1.86 (1.26–2.74)	0.002	1.77 (1.20–2.61)	0.004
	**DU**	1.20 (0.87–1.65)	0.266	1.00 (0.69–1.43)	0.982	0.95 (0.66–1.37)	0.800
	**DM**						
	**Normal**	Reference	-	Reference	-	Reference	-
	**GERD**	2.16 (1.48–3.17)	<0.001	1.73 (1.18–2.55)	0.005	1.68 (1.14–2.48)	0.009
	**GU**	1.83 (0.98–3.41)	0.056	1.39 (0.74–2.61)	0.308	1.32 (0.70–2.49)	0.386
	**DU**	2.47 (1.46–4.18)	<0.001	2.18 (1.27–3.72)	0.005	2.10 (1.22–3.59)	0.007
**Female**	**MetS**						
	**Normal**	Reference	-	Reference	-	Reference	
	**GERD**	1.23 (0.99–1.52)	0.059	0.91 (0.71–1.17)	0.467	0.91 (0.71–1.17)	0.909
	**GU**	0.94 (0.56–1.56)	0.808	0.58 (0.32–1.08)	0.087	0.58 (0.31–1.08)	0.580
	**DU**	1.06 (0.66–1.69)	0.822	0.66 (0.39–1.15)	0.142	0.66 (0.38–1.14)	0.658
	**DM**						
	**Normal**	Reference	-	Reference	-	Reference	
	**GERD**	0.98 (0.64–1.51)	0.928	0.71 (0.45–1.12)	0.136	0.71 (0.45–1.12)	0.143
	**GU**	1.29 (0.53–3.14)	0.576	0.87 (0.34–2.23)	0.768	0.89 (0.34–2.28)	0.801
	**DU**	2.18 (1.08–4.40)	0.030	1.64 (0.78–3.48)	0.196	1.68 (0.79–3.56)	0.180

^a^ Adjusted covariates

Model 1 = unadjusted

Model 2 = Model 1 + age, BMI, LDL-C, hsCRP

Model 3 = Model 2 + history of smoking, drinking

### HP infection and the risks of incident MetS and DM

After a period of follow-up, a longitudinal analysis was conducted by the multivariable Cox proportional hazard model to examine whether HP infection could predict the risks of developing incident cardiometabolic disease, and the results are shown in Table 4. It was surprising that HP infection could only predict the risk for developing incident DM in males with an HR of 1.55 (95%CI = 1.15–2.10) after fully adjusting for pertinent covariables.

**Table 4:** Association between HP infection and the risks of developing incident MetS and DM categorized by gender.

	Sex	Model ^a^ 1 HR (95% CI)	*P* Value	Model ^a^ 2 HR (95% CI)	*P* Value	Model ^a^ 3 HR (95% CI)	*P* Value
	**MetS**						
**HP infection**	**Male**	1.15 (0.97–1.36)	0.104	1.10 (0.93–1.31)	0.268	1.09 (0.92–1.30)	0.307
	**Female**	1.14 (0.89–1.47)	0.299	0.87 (0.67–1.12)	0.275	0.87 (0.68–1.13)	0.298
	**DM**						
**HP infection**	**Male**	1.75 (1.29–2.36)	<0.001	1.58 (1.17–2.15)	0.003	1.55 (1.15–2.10)	0.005
	**Female**	0.98 (0.56–1.69)	0.928	0.74 (0.43–1.29)	0.294	0.74 (0.43–1.29)	0.290

^a^ Adjusted covariates

Model 1 = unadjusted

Model 2 = Model 1 + age, BMI, LDL-C, hsCRP

Model 3 = Model 2 + history of smoking, drinking

### HP infection and the risks of incident MetS and DM with endoscopy findings

In Table 5, endoscopy findings of GERD in male subjects were predictive for incident MetS and DM with HRs of 1.21 (95%CI = 1.00–1.46) and 1.53 (95%CI = 1.01–2.31), respectively. Endoscopy findings of GU had a higher tendency to predict the risk of incident DM with an HR of 1.55 (95%CI = 1.15–2.10). No significant association was found in the female population.

**Table 5:** Association between HP infection and the risks of developing incident MetS and DM with endoscopy findings categorized by gender.

	Variable	Number	Person Years	Model ^a^ 1 HR (95% CI)	*P* Value	Model ^a^ 2 HR (95% CI)	*P* Value	Model ^a^ 3 HR (95% CI)	*P* Value
**GERD**									
**HP** **Infection**	**MetS**								
	**Male**	1820	956.37	1.31 (1.09–1.58)	0.004	1.23 (1.03–1.49)	0.027	1.21 (1.00–1.46)	0.047
	**Female**	1270	402.67	1.12 (0.92–1.37)	0.271	0.99 (0.81–1.22)	0.931	0.99 (0.81–1.22)	0.956
	**DM**								
	**Male**	1820	956.37	2.01 (1.34–3.02)	<0.001	1.59 (1.06–2.40)	0.027	1.53 (1.01–2.31)	0.043
	**Female**	1270	402.67	0.98 (0.64–1.50)	0.919	0.83 (0.54–1.28)	0.389	0.83 (0.54–1.28)	0.401
**Gastric ulcer**									
**HP** **Infection**	**MetS**								
	**Male**	164	90.75	1.72 (1.27–2.32)	<0.001	1.59 (1.18–2.15)	0.003	1.55 (1.15–2.10)	0.004
	**Female**	115	43.97	0.94 (0.57–1.53)	0.793	0.72 (0.44–1.18)	0.190	0.71 (0.44–1.17)	0.177
	**DM**								
	**Male**	164	90.75	1.72 (0.86–3.47)	0.127	1.34 (0.66–2.70)	0.418	1.27 (0.63–2.57)	0.499
	**Female**	115	43.97	1.28 (0.54–3.04)	0.573	0.87 (0.37–2.09)	0.763	0.87 (0.37–2.14)	0.799
**Duodenal ulcer**									
**HP** **Infection**	**MetS**								
	**Male**	261	201.80	1.08 (0.80–1.45)	0.634	1.01 (0.75–1.36)	0.941	0.99 (0.73–1.33)	0.933
	**Female**	170	50.83	1.00 (0.64–1.56)	0.997	0.85 (0.54–1.33)	0.485	0.85 (0.54–1.34)	0.486
	**DM**								
	**Male**	261	201.80	1.95 (1.11–3.44)	0.011	1.74 (0.99–3.07)	0.055	1.62 (0.91–2.87)	0.098
	**Female**	170	50.83	1.72 (0.83–3.58)	0.145	1.35 (0.65–2.81)	0.427	1.37 (0.65–2.86)	0.408

^a^ Adjusted covariates

Model 1 = unadjusted

Model 2 = Model 1 + age, BMI, LDL-C, hsCRP

Model 3 = Model 2 + history of smoking, drinking

## Discussion

In the present study, we proposed that HP infection was significantly associated with MetS and DM only in male subjects and that those with different endoscopic findings also shown a correlation with cardiometabolic diseases in a cross-sectional analysis. After a period of follow-up, our findings highlight the likelihood that participants with HP infection had high risks of developing incident DM. In addition, endoscopic findings of GERD and DU also predictive of incident DM. To the best of our knowledge, our study was the first to examine the role of HP infection and endoscopy findings in predicting incident DM in a large population-based longitudinal study of the general population.

Accumulating evidence has suggested HP infection as a risk factor for developing DM. A positive association between HP infection and the prevalence of DM was noted in a cross-sectional study[6]. HP infection was more prevalent in patients with DM compared to controls, and the presence of HP infection was significantly correlated with the level of HbA1C[15]. Kayar et al. showed a significant relationship between HP infection and MetS, insulin resistance, inflammation, and diabetic complications[9]. Recently, seropositivity of HP infection was reported to be associated with lower risk of DM[16]. However, the concentrations of antibodies against CagA, a major factor in HP infection, might be elevated in patients who had a negative HP serological test[11]. A negative result does not rule out the possibility of a previous exposure to HP infection, and these findings may have resulted in a statistical error. In addition, information on HP eradication treatments were not collected in that study, which might have underestimated the protective ability of HP infection in DM. Our findings were consistent with the above results, and we demonstrated a significant relationship between HP infection and increased risk of DM in a prospective cohort study. Although no concrete evidence demonstrated that HP infection played an important role in DM, there were several plausible pathogenic mechanisms to implicate HP infection and elucidate this issue. HP affected the immune response of the host organism by pathways such as CagA and VacA[17]. VacA led to various disturbances such as cell vacuolation, autophagy and inhibition of T-cell proliferation[18]. Multiple immune factors including interleukin (IL)-1β, IL-6 and tumor necrosis factor (TNF)-α induced by HP infection contributed to gastric mucosal inflammation and a general systemic pro-inflammatory state[19]. IL-8 induction was a typical event in HP infection, as was simultaneous induction of production of other endogenous cytokines[20]. Those inflammatory markers were correlated with insulin resistance and development of DM[21]. Adipose tissue inflammation was considered a key factor in the pathogenesis of insulin resistance and potential *β*-cell-related autoinflammation, which disturbed insulin secretion in DM[22]. Gastritis induced by HP infection could affect the secretion of gastric-related hormones such as leptin, ghrelin, gastrin and somatostatin, which might influence a predisposition to DM[23].

The close association between GU and DU diseases with HP infection has been examined for decades. An infected individual has a higher lifetime risk for developing peptic ulcer disease than a noninfected subject[24]. The precise mechanism by which HP infection leads to peptic ulcer is incompletely understood. However, the bacterium appears to affect several aspects of intestinal and mucosal physiology to cause the diseases. Nomura et al. suggested that preexisting HP infection increased the risk for subsequent development of either GU and DU[25]. Chronic HP infection contributed to increased basal and stimulated acid output, particularly in patients who developed DU[26].

The association between HP infection and DM was male predominant in our study.

This finding was in line with a previous study in which inflammation and activity scores in the antrum with HP infection were higher in males[27]. According to a large dataset including worldwide mortality and prevalence of cancers, the incidence rate of gastric cancer in males was approximately 2-fold higher than that in females[28]. In a mouse model research, Ohtani et al. confirmed that severe dysplasia and gastric carcinogenesis were male predominant in HP-infected subjects[29]. Ovarian-dependent female hormones, especially E2, provided a protective role against gastric carcinogenesis and inflammation of gastric tissue in HP-infected INS-GAS mice[30]. Exogenous E2 supplementation showed a protective effect against the development of HP-induced gastritis and premalignant lesions via several mechanisms such as stimulation of IL-10 activity, enhancement of Th2-mediated immune responses, and inhibitory effects on epithelial cell proliferation[30]. Sex differences in gastric mucosal responses to HP infection were proposed that might be correlated with sex differences in the incidence of stomach cancer[27]. Reduced expression of TFF1 mRNA, a defensive factor in the gastrointestinal mucosa, was caused by HP infection and led to inflammatory response and precursory gastric lesions[31]. Based on the above studies, the sex difference among the association of HP infection with DM in the present study might be related to sex hormone induced-inflammation.

The strengths of our study were that first, participants were obtained from a large population-based survey. Furthermore, a longitudinal analysis was performed to assess the causal association between HP infection and risks of DM. Next, the diagnosis of HP infection during the study was conducted by endoscopic examinations with RUTs. Several lines of evidence have supported that the RUT remain the uncontested gold standard for diagnosing HP infection[32]. Despite the advantages mentioned above, there were still potential limitations in our study. First, the study sample was composed of the general population from a single medical institution that limited ethnic diversity in the participants, which might influence the results which regard to racial differences. Second, the present study was retrospective, and these findings should be clarified further by large-scale prospective studies. Next, the information on social economic status was not available in the health examination. Future study should include this potential covariable for adjustment. Finally, only one test was performed in the health examinations. The prevalence and strains of HP based on endemic areas, accessibility and clinical situations should be considered to select a better test for each patient. Combining the results of at least two tests could be a reasonable strategy in routine clinical practice to obtain the most reliable result[11].

In summary, an association between HP infection and DM was demonstrated in our study that might occur through several mechanisms such as inflammation and immune response. Notably, participants with DUs had a high risk of incident DM after a period of follow-up. Preexisting HP infection might result in endocrinological derangements and inflammation that harbor a predisposing milieu for incident MetS and DM. Further randomized control trials investigating molecular mechanisms for the suppression of HP infection could contribute to the development of novel therapeutic strategies applicable to peptic diseases and DM.
